# SARS-CoV-2 ORF3a-Mediated NF-κB Activation Is Not Dependent on TRAF-Binding Sequence

**DOI:** 10.3390/v15112229

**Published:** 2023-11-08

**Authors:** Brianna M. Busscher, Henock B. Befekadu, Zhonghua Liu, Tsan Sam Xiao

**Affiliations:** 1 Department of Pathology, School of Medicine, Case Western Reserve University, Cleveland, OH 44106, USA; brianna.busscher@case.edu (B.M.B.); zhonghua.liu2@case.edu (Z.L.); 2 Department of Physiology and Biophysics, School of Medicine, Case Western Reserve University, Cleveland, OH 44106, USA; henock.befekadu@case.edu; 3 MOE Key Laboratory for Membraneless Organelles and Cellular Dynamics, Hefei National Research Center for Physical Sciences at the Microscale, Division of Life Sciences and Medicine, University of Science and Technology of China, Hefei 230027, China

**Keywords:** SARS-CoV-2 ORF3a, NF-κB, TRAF proteins, inflammation

## Abstract

Severe Acute Respiratory Syndrome Coronavirus 2 (SARS-CoV-2) has caused a global pandemic of Coronavirus Disease 2019 (COVID-19). Excessive inflammation is a hallmark of severe COVID-19, and several proteins encoded in the SARS-CoV-2 genome are capable of stimulating inflammatory pathways. Among these, the accessory protein open reading frame 3a (ORF3a) has been implicated in COVID-19 pathology. Here we investigated the roles of ORF3a in binding to TNF receptor-associated factor (TRAF) proteins and inducing nuclear factor kappa B (NF-κB) activation. X-ray crystallography and a fluorescence polarization assay revealed low-affinity binding between an ORF3a N-terminal peptide and TRAFs, and a dual-luciferase assay demonstrated NF-κB activation by ORF3a. Nonetheless, mutation of the N-terminal TRAF-binding sequence PIQAS in ORF3a did not significantly diminish NF-κB activation in our assay. Our results thus suggest that the SARS-CoV-2 protein may activate NF-κB through alternative mechanisms.

## 1. Introduction

Severe Acute Respiratory Syndrome Coronavirus 2 (SARS-CoV-2) has infected over three quarters of a billion people, millions of whom succumbed to Coronavirus Disease 2019 (COVID-19) or associated complications [1]. COVID-19 is characterized by an overabundant inflammatory response that can progress to cytokine storm and cause severe tissue damage, especially in the lungs, but the pathogenesis of the disease is still poorly understood [2,3,4]. While much attention has been given to the SARS-CoV-2 structural proteins such as Spike, the SARS-CoV-2 genome encodes sixteen non-structural proteins and nine accessory proteins that may each play important roles in the development of COVID-19 [5]. The accessory protein open reading frame 3a (ORF3a) in particular was reported to be critical to disease pathology in a mouse model of SARS-CoV-2 infection [6]. In that study, mice infected with virus lacking ORF3a had a lower mortality rate (25% by day 15) than those infected with wildtype virus (100% by day 6), and ORF3a deletion was correlated with lower viral titer in the lungs of the infected mice [6]. In another study, ORF3a expression in brains of mice led to neuroinflammation and neurodegeneration [7].

ORF3a is a transmembrane protein capable of forming homodimers and potentially homotetramers. It is also encoded in the SARS-CoV genome [8,9], and the two ORF3a proteins have high homology in primary structure with 73% sequence identity, as well as in secondary, tertiary, and quaternary structures reported in multiple cryo-EM studies of SARS-CoV ORF3a (PDB ID: 8EQS) and SARS-CoV-2 ORF3a (8EQJ, 8EQT, and 7KJR) [8,10]. ORF3a has many proposed functions, such as inducing cell death through apoptosis or inflammasome activation, leading to pyroptosis [7,11,12,13,14,15,16,17,18], disrupting cellular membrane structures [11,15,18,19], and impairing autophagy [7,18,19,20,21,22,23,24]. ORF3a dimers have also been thought to form a cation channel [8,9,16,25]. However, recent studies have challenged this hypothesis, noting that the proposed pore is not suitable for cation permeation based on the cryo-EM structures [10,26]. Instead, the previously observed ion conductance may be due to ORF3a-induced pannexin channel activities [26]. 

Another function of ORF3a, as reported for the SARS-CoV protein, was the activation of the NLRP3 inflammasome [16,17]. Canonical inflammasome activation requires a priming step, which activates NF-κB to upregulate expression of the sensor proteins such as NLRP3, pro-caspase-1, and pro-IL-1β. This is followed by an assembly step in which NLRP3 oligomerizes, pro-caspase-1 is recruited and auto-processed, and mature caspase-1 cleaves gasdermin-D, pro-IL-1β, and pro-IL-18 [27,28]. SARS-CoV ORF3a can activate NF-κB at the priming step, and this effect was shown to be impaired upon mutation of three residues (P36, Q38, S40) in its N-terminal Pro-Leu-Gln-Ala-Ser (PLQAS) sequence that conforms to TNF receptor-associated factor (TRAF)-binding consensus sequences [17,29]. In agreement, wildtype SARS-CoV ORF3a could be co-immunoprecipitated with multiple TRAF proteins such as TRAF2, TRAF3, and TRAF6 [17]. The TRAF proteins participate in multiple NF-κB signaling pathways, functioning as adaptor proteins and E3 ubiquitin ligases. They form trimers and interact with receptor intracellular domains and other proteins through their C-terminal TRAF-C domains [29,30,31]. Since the putative TRAF-binding sequence in ORF3a is conserved between SARS-CoV (PLQAS) and SARS-CoV-2 (PIQAS), and generally conforms to the TRAF-binding consensus sequences [29], we hypothesized that SARS-CoV-2 ORF3a might also bind to TRAFs and thereby induce NF-κB activation.

To investigate this, we solved the structures of the TRAF-C domains from TRAF2 and TRAF3 in complex with a SARS-CoV-2 ORF3a peptide harboring the TRAF-binding sequence. The structures confirm that the TRAF-binding sequence from ORF3a indeed mediated its binding to the TRAF-C domains, and such binding was of low affinity as revealed by fluorescence polarization (FP) assays. Using dual-luciferase experiments measuring NF-κB activity, we found that SARS-CoV-2 ORF3a activated NF-κB compared with controls, but mutation of the TRAF-binding sequence did not significantly alter ORF3a-induced NF-κB activation. Our results thus suggest that in contrast to previous studies on ORF3a from SARS-CoV, the SARS-CoV-2 protein may activate NF-κB through alternative mechanisms.

## 2. Materials and Methods

### 2.1. Cells, Antibodies, Plasmids, and Peptides

Cells. HEK293T cells were grown in DMEM (Gibco, Dublin, Ireland) supplemented with 10% fetal bovine serum (Gibco) and 1% penicillin/streptomycin (Gibco). 

Antibodies. Anti-SARS-CoV-2 ORF3a antibody (Cell Signaling Technology (Danvers, MA, USA), #34340S) was used at 1:1000 dilution and anti-GAPDH antibody (Santa Cruz, sc-47724) was used at 1:1000 dilution for Western blotting. 

Plasmids. The Promega firefly luciferase plasmid, pRL-TK Renilla luciferase plasmid (Promega, Madison, WI, USA), and pBZ vector were gifts from Dr. Derek Abbott at Case Western Reserve University. The SARS-CoV-2 ORF3a plasmid was a gift from Dr. David Ho at Columbia University. 

Peptides. Peptides were synthesized by GenScript (Piscataway, NJ, USA). Peptides used for FP assays were modified with 5-carboxyfluorescein (5-FAM) followed by a 6-aminohexanoic acid (Ahx) linker at the N-terminus and amidation at the C-terminus. Peptides lacking the 5-FAM-Ahx modification were acetylated at the N-terminus and used in crystallization. Peptide sequences are listed below.

5-amino acid (5-aa) ORF3a peptide: 36-PIQAS-40
9-aa ORF3a peptide: 34-TIPIQASLP-42
16-aa ORF3a peptide: 30-RATATIPIQASLPFGW-45
Selenomethionine (Se-Met) ORF3a peptide: 30-RATATIP[Se-Met]QASLPFGW-45

### 2.2. Protein Expression and Purification

*E. coli* strain BL21 (DE3) Codon Plus RIPL (Agilent Technologies, Santa Clare, CA, USA) was used for recombinant protein expression. TRAF-C domains of human TRAF2 (Uniprot: Q12933, residues 315–501) and human TRAF3 (Uniprot: Q13114, residues 341–568 or 377–568) were expressed as 6xHis-Small ubiquitin-like modifier (SUMO) N-terminal fusion proteins in pSMT3 vector. Protein expression was induced by addition of isopropyl ß-D-1-thiogalactopyranoside (IPTG) (GoldBio, St. Louis, MO, USA) at a final concentration of 0.5 mM for 24 h at 18 °C. Bacteria were harvested by centrifugation at 5000 rpm and 20 °C for 15 min. Pelleted cells were resuspended with 40 mL of lysis buffer (25 mM Tris pH 7.5, 150 mM NaCl, 5 mM imidazole, 5 mM β-mercaptoethanol (β-ME)) per 1 L of starting culture. Resuspended cells were then sonicated, and lysate was ultracentrifuged at 25,000 rpm at 4 °C for 35 min. The soluble fraction was applied to Ni-NTA agarose resin (MCLab, South San Francisco, CA, USA). The loaded column was washed with lysis buffer and subsequently with lysis buffer containing 10 mM imidazole and 20 mM imidazole. 

The bound protein was eluted from the column using an elution buffer (25 mM Tris pH 7.5, 150 mM NaCl, 0.4 M imidazole, 5 mM β-ME) and dialyzed in the lysis buffer. The fusion protein samples were used in the fluorescence polarization experiments due to improved solubility and stability at higher concentrations. For crystallization, the eluted fusion protein samples were incubated with SUMO protease Ulp1 to cleave the 6xHis-SUMO tag. The cleavage mixture was loaded onto a second Ni-NTA column, and the flow-through was collected and concentrated. To further truncate the TRAF3 protein to facilitate crystallization, the sample was digested with trypsin (Sigma-Aldrich, St. Louis, MO, USA) at a molar ratio of 1:750 (enzyme: protein) for 4 h at 4 °C [32]. The reaction was terminated with 1 mM of phenylmethylsulfonyl fluoride (PMSF) (Biosynth, Staad, Switzerland). The TRAF2 protein and truncated TRAF3 samples were further purified through size exclusion chromatography in a buffer containing 25 mM Tris pH 7.5 and 150 mM NaCl.

### 2.3. Protein Crystallization and Structural Determination

Sparse matrix crystallization screening was performed for the purified TRAF-C domains from TRAF2 and TRAF3 using reagents from Molecular Dimensions (Holland, OH, USA). The 16-aa ORF3a peptide or Se-Met ORF3a peptide was added to the concentrated protein samples at a molar ratio of 5:1 (peptide:protein) prior to setting up crystal drops using the sitting drop method. The TRAF-C domain from TRAF2 was crystallized at 4 °C with a reservoir solution containing 20% PEG8000 plus 0.1 M sodium citrate pH 5.0, or 20% PEG8000, 0.2 M ammonium sulfate, plus 0.1 M MES pH 6.5. The TRAF-C domain from TRAF3 was crystallized at 20 °C with a reservoir solution containing 13% PEG 3350, 0.25 M ammonium sulfate, plus 0.1 M Tris pH 8.0. Crystals were preserved in a cryoprotectant solution containing 20% glycerol and 1 mM ORF3a peptides.

X-ray diffraction data were collected at beamlines NE-CAT (24-ID) and GM/CAT (23-ID) at the Advanced Photon Source, Argonne National Laboratory, and processed with program XDS [33]. The structure was determined using the human TRAF2 (PDB: 1D01) or TRAF3 (PDB: 4GHU) structures as search models. Manual model building and molecular replacement/refinement were performed with Coot [34] and Phenix [35], respectively. The crystal structures were validated by the MolProbity server [36]. Figures were prepared using PyMol (The PyMOL Molecular Graphics System, Version 1.8.2.3 Schrödinger, LLC.).

### 2.4. Fluorescence Polarization

Recombinant 6xHis-SUMO-TRAF2 (residues 315–501) or 6xHis-SUMO-TRAF3 (residues 341–568) was incubated in a 96-well plate for 10 min with a buffer containing 25 mM Tris pH 8.0, 150 mM NaCl, 1 mM DTT, 1 mM EDTA plus 30 nM or 50 nM of fluorescent 5-FAM-Ahx-PIQAS peptide. Peptide alone (without TRAF protein) was incubated in the buffer as a control. Triplicate wells were used for each concentration of the TRAF protein. Fluorescence polarization was measured using a SpectraMax i3x plate reader (Molecular Devices, San Jose, CA, USA) in technical triplicates at 10, 30, 60, and 90 min, using 485 nm and 535 nm as excitation and emission wavelengths, respectively. Technical replicates for each time point were averaged, and the lowest average FP reading was subtracted from the average reading for each TRAF concentration. Data were plotted and analyzed with GraphPad Prism (GraphPad Software v.10.0, San Diego, CA, USA) to fit with a non-linear binding function expressed as: Y = Bmax × X/(Kd + X), where Bmax is the maximum specific bindings and Kd is the equilibrium binding constant. 

### 2.5. NF-κB Dual Luciferase Reporter Assay

Sample preparation. HEK293T cells were seeded in 12-well plates and transfected 24 h later in triplicates with 0.7 μg of Promega firefly luciferase vector, 0.07 μg of pRL-TK Renilla luciferase vector, plus up to 5 μg of either pBZ empty vector, ORF3a WT or TRAF-mut ORF3a pBZ plasmid per well using calcium phosphate or the polyethylenimine (PEI) transfection method. Media was changed 5 h post-transfection. Six hours before harvesting, positive control cells were treated with 5–10 ng/mL of recombinant human TNF-⍺ (Gibco, Waltham, MA, USA). At 48 h post transfection, cells were harvested using Passive Lysis Buffer (Promega, Madison, WI, USA), sonicated, centrifuged, and stored at −80 °C until the assay was performed. 

Assay. Dual-Luciferase Reporter Assay kit (Promega, Madison, WI, USA) reagents were prepared according to the manufacturer’s protocol. A quantity of 15 μL of cell lysate was mixed with 50 μL of Luciferase Assay Reagent II (LAR II) in a 96-well plate, and firefly luciferase activity was measured using a GloMax Discover plate reader (Promega, Madison, WI, USA). A quantity of 50 μL of Stop&Glo reagent was then added, and the Renilla luciferase activity was measured. Results were reported as the ratio of Firefly/Renilla luciferase activities and plotted and analyzed with GraphPad Prism.

## 3. Results

### 3.1. Crystal Structure of an ORF3a Peptide in Complex with the TRAF-C Domains

**Figure 1:**
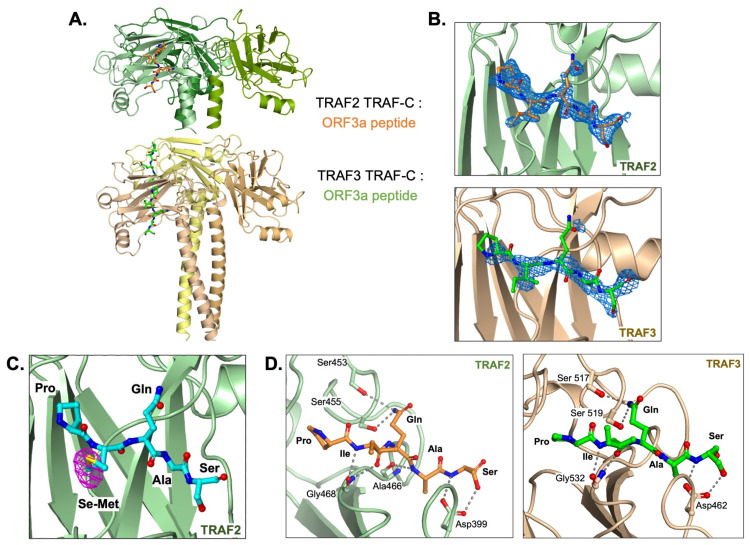
TRAF2 and TRAF3 TRAF-C domains in complex with ORF3a peptide. (**A**) Trimer structure of TRAF2 TRAF-C (**upper**) or TRAF3 TRAF-C (**lower**) in complex with one or two ORF3a peptides, respectively. Five peptide residues, PIQAS, are shown as orange or green ball-and-stick models in the TRAF2 and TRAF3 structures, respectively. (**B**) Fo-Fc omit map (blue mesh, 2.5 sigma) in the peptide-binding groove of TRAF2 (**upper panel**) or TRAF3 (**lower panel**) with PIQAS residues from the ORF3a peptide superimposed. (**C**) Residues from selenomethionine (Se-Met)-containing ORF3a peptide in complex with the TRAF2 TRAF-C domain. Anomalous difference map (magenta mesh) is shown at 4 sigma. (**D**) Hydrogen bonds between residues in ORF3a peptide and residues in TRAF2 (**left**) or TRAF3 (**right**) TRAF-C domains are shown as gray dotted lines.

To investigate whether the SARS-CoV-2 ORF3a N-terminal region containing the PIQAS sequence is capable of binding to TRAF molecules, we crystallized the TRAF2 TRAF-C domain and the TRAF3 TRAF-C domain in complex with a 16-amino acid peptide from the N-terminal region of ORF3a (residues 30–45). X-ray diffraction data were collected up to 1.9 Å and 2.5 Å resolutions for the TRAF2:ORF3a peptide complex and TRAF3:ORF3a peptide complex, respectively (Table 1, PDB ID codes 8T5Q and 8T5P, respectively). The crystallographic asymmetric unit in the TRAF2 crystal lattice contains three TRAF-C molecules forming a trimer, while the asymmetric unit in the TRAF3 crystal lattice contains six TRAF-C molecules forming two trimers. We observed density for five residues (PIQAS) of the ORF3a peptide, which binds at a solvent-facing groove in the globular region of the TRAF-C domain (Figure 1A). The TRAF-C binding sites are not fully occupied—in our model, each TRAF trimer has either one or two peptides bound, which is similar to previously reported TRAF:peptide complex structures. The omit map (Fo-Fc) at 2.5 sigma for one peptide from each structure is shown in Figure 1B. We also co-crystallized TRAF2 TRAF-C with an ORF3a peptide in which the isoleucine was replaced with selenomethionine and collected anomalous diffraction data. The anomalous difference density map at 4 sigma (Figure 1C) clearly shows the position of the heavy selenium atom, which further confirms the position of the PIQAS peptide bound at the TRAF-C domain groove.

The ORF3a peptide forms several main chain and side chain hydrogen bonds with the surrounding residues from TRAF2 or TRAF3 (Figure 1D). Of note, the side chain from Q38 in the peptide forms multiple hydrogen bonds with two serine residues in the TRAF-C domain. Its capacity to form these bonds and thus stabilize the ORF3a-TRAF interaction is consistent with the conservation of this residue in previously published TRAF-binding peptides [29]. The S40 side chain is also within hydrogen bonding distance of TRAF residues that may contribute to peptide binding. Additionally, the P36 residue, also highly conserved among TRAF2/3-binding peptides, is in proximity to other hydrophobic and aromatic residues in the TRAF molecule, particularly F511, with which it may form hydrophobic interactions. Overall, several hydrogen bonds and hydrophobic interactions contribute to ORF3a interaction with TRAF2 or TRAF3 through the five conserved residues. 

### 3.2. ORF3a Peptide Binds to TRAF-C Domains with Low Affinity

**Figure 2:**
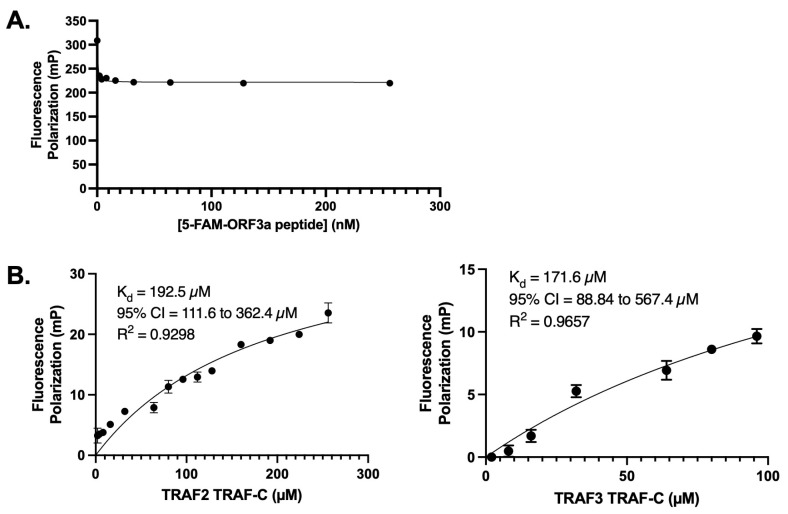
Fluorescence polarization (FP) assay with 5-FAM-ORF3a peptide and TRAF2 or TRAF3 TRAF-C domain. (**A**) FP assay with 5-FAM-ORF3a peptide alone (0–256 nM) to determine optimum concentrations for experiments with TRAF proteins. (**B**) Nonlinear regression curves generated from FP assays. **Left:** Binding of 0–256 μM of TRAF2 TRAF-C domain and 30 nM of 5-FAM-ORF3a peptide. Two independent experiments conducted in triplicate. **Right:** FP assay with 0–96 μM of TRAF3 TRAF-C domain and 50 nM of 5-FAM-ORF3a peptide. Three independent experiments conducted in triplicate. Plots generated and statistics calculated in GraphPad Prism. The error bars represent standard deviation (SD).

To quantify the strength of the ORF3a-TRAF interaction, we performed fluorescence polarization (FP) experiments with fluorescently labeled PIQAS peptide (Figure 2). To enhance TRAF protein stability at high concentrations in the assay, SUMO fusion proteins of the TRAF-C domains from TRAF2 or TRAF3 were used. The concentration of the TRAF proteins used in the assays ranged from 0–256 μM for TRAF2 and 0–96 μM for TRAF3. A constant peptide concentration (30 nM peptide for TRAF2 experiments and 50 nM peptide for TRAF3 experiments) was selected based on FP curves generated using ORF3a peptide alone with the aim of minimizing background noise while maintaining significant FP signals (Figure 2A). The binding affinities between the PIQAS peptide and SUMO-TRAF-C proteins were in the range of 150–200 μM (192.5 μM for TRAF2 and 171.6 μM for TRAF3) (Figure 2B). This is consistent with previously reported affinity measurements of ~200 μM for TRAF proteins binding to peptides from various membrane receptors [37].

### 3.3. ORF3a-Mediated NF-κB Activation Does Not Depend on Its TRAF-Binding Sequence

**Figure 3:**
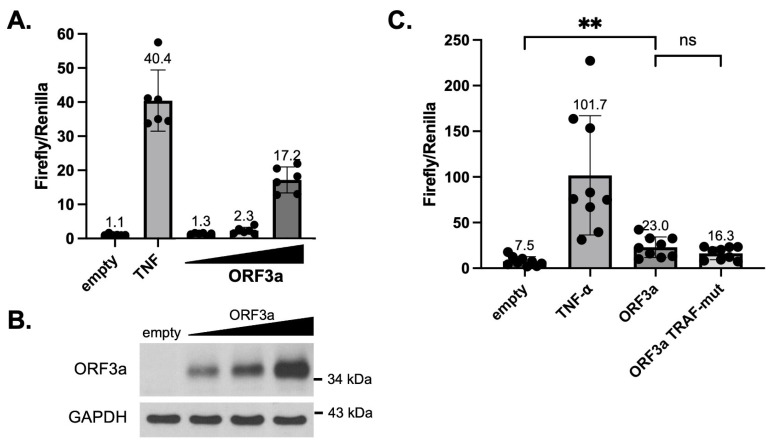
SARS-CoV-2 ORF3a-mediated NF-κB activation. (**A**) HEK293T cells were transfected with NF-κB responsive firefly luciferase plasmid and constitutive Renilla luciferase plasmid, plus empty vector or increasing amounts of SARS-CoV-2 ORF3a-expressing plasmid (1.7, 3.3, or 5 μg per well in 12-well plates). Lysates were harvested 48 h after transfection and used in a dual-luciferase assay. The ratio of firefly to Renilla luciferase activity was plotted. As a positive control, cells were treated with 5 ng/mL of TNF-⍺ for 6 h prior to harvesting. (**B**) SARS-CoV-2 ORF3a in HEK293T dual-luciferase samples was detected by immunoblotting with anti-SARS-CoV-2 ORF3a antibody. Detection of GAPDH was used as a loading control. (**C**) HEK293T cells were transfected with NF-κB responsive firefly luciferase plasmid and constitutive Renilla luciferase plasmid, plus empty vector or plasmids expressing wildtype or TRAF-mut ORF3a. Lysates were harvested 48 h after transfection and assays were performed similar to (**A**). *p*-values: empty vs. ORF3a = 0.0017, ORF3a vs. ORF3a TRAF-mut (ns) = 0.145. ** *p* < 0.01. Plots were generated and statistics were calculated in GraphPad Prism. The error bars represent standard deviation (SD).

Because ORF3a was shown to activate NF-κB in multiple studies and mutation of the TRAF-binding sequence in SARS-CoV ORF3a ablated NF-κB activation [17,18,38,39], we investigated the effect of mutating the TRAF-binding sequence on SARS-CoV-2 ORF3a-mediated NF-κB activation. HEK293T cells were transfected with firefly and Renilla luciferase plasmids, plus wildtype (WT) ORF3a plasmid or mutant (TRAF-mut) ORF3a plasmid. In the TRAF-mut ORF3a plasmid, the TRAF-binding sequence was mutated to alanine residues (PIQAS to AAAAA) similar to previous studies of SARS-CoV ORF3a [17]. As the PIQAS is located at a loop region exposed to the solvent, such mutation to Ala is unlikely to affect the folding or structure of ORF3a. Our data confirmed previously published findings [38,39] that SARS-CoV-2 ORF3a expression activated NF-κB to a moderate extent (2- to 4-fold above the empty vector) and NF-κB activation increases with increasing ORF3a expression (Figure 3A,B). We also found that TRAF-mut ORF3a activated NF-κB to a similar extent as WT ORF3a (Figure 3C), indicating that the TRAF-binding sequence does not make a significant contribution to NF-κB activation by SARS-CoV-2 ORF3a.

## 4. Discussion

ORF3a is an important accessory protein in the SARS-CoV-2 genome, and studying its functions may help elucidate the pathologic mechanisms that lead to COVID-19. We investigated the ability of ORF3a to activate NF-κB as a priming step for canonical inflammasome activation and a potential source of inflammation during SARS-CoV-2 infection. The important role of TRAF proteins in NF-κB signaling is well established [30,31,40,41,42], and SARS-CoV ORF3a was reported to bind to TRAFs [17]. Both SARS-CoV and SARS-CoV-2 ORF3a contain a sequence within their N-terminal domain that resembles other peptides from host proteins that bind TRAF2 or TRAF3 [29]. We therefore assessed the ability of a peptide containing a putative TRAF-binding sequence (PIQAS) from SARS-CoV-2 ORF3a to bind to TRAF2 or TRAF3. 

Crystal structures revealed that a PIQAS-containing peptide can bind to TRAF2 or TRAF3 at the previously identified peptide-binding surface in the TRAF-C domain. Using fluorescence polarization assays, we calculated the affinity of this interaction to be 150–200 μM, which is similar to the affinity of other TRAF-binding peptides in complex with the TRAF-C domain [37]. Our data thus suggest low affinity interaction between ORF3a and TRAF2/3, which does not take into account potential multivalent interactions between ORF3a dimers or tetramers and TRAF trimers. Such potential avidity effects may increase the strength of their interaction during SARS-CoV-2 infection beyond our measured affinity. 

The published structures of ORF3a place its N-terminal domain in extracellular or luminal compartments and its C-terminal domain in the cytosolic compartment. This topology is supported by the positive-inside rule, which describes the tendency of loops between transmembrane helices to face the cytosol when they contain positively charged residues (Arg and Lys) because of the negative potential of the cytosol relative to the extracellular space [43]. However, ORF3a localizes not only to the plasma membrane but also to intracellular membranes (lysosomes, endosomes, Golgi, endoplasmic reticulum) [9,11,13,44,45,46,47], and while there is experimental evidence to support the proposed ORF3a topology at the plasma membrane [48], it is unclear whether its orientation in intracellular membranes is the same. Additionally, ORF3a has been reported to disrupt and reorganize membrane structures in infected cells or when overexpressed [11,15,18,19]. Yue and colleagues found that SARS-CoV ORF3a expression in HeLa cells leads to permeabilization of the lysosomal membrane and release of lysosomal cathepsins into the cytosol [15], which may facilitate the exposure of cytosolic proteins to membrane-bound ORF3a on either side of the lysosomal membrane. Thus, the disruption of membrane structures by ORF3a may contribute to its interaction with various signaling proteins independently of its topology. 

Our data also revealed that SARS-CoV-2 ORF3a can activate NF-κB, which appears to be independent of the PIQAS sequence. These results differ from the reported activation of NF-κB by SARS-CoV ORF3a, which required its TRAF-binding sequence PLQAS [17]. As the only different residues (Ile from SARS-CoV-2 ORF3a and Leu from SARS-CoV) are both hydrophobic residues of similar size, their modes of interaction with partner proteins are expected to be similar. This suggests that ORF3a from SARS-CoV and SARS-CoV-2 may activate NF-κB via different mechanisms, with the latter requiring sequences outside of the TRAF-binding sequence. Indeed, Nie and colleagues recently published evidence that SARS-CoV-2 ORF3a interacts with NEMO and IKKβ to stabilize the NEMO-IKKβ complex and thereby activate NF-κB [39]. This complex is downstream of TRAFs in the NF-κB activation pathway [40,41], which may explain why the TRAF-binding sequence was not strictly required for the SARS-CoV-2 ORF3a-mediated NF-κB activation in our study.

We note that the scope of our current study is limited to NF-κB activation by SARS-CoV-2 ORF3a. ORF3a is reported to affect multiple signaling pathways including apoptosis, autophagy, and inflammasomes, in addition to modulating cellular membrane structures and forming cation channels [7,8,9,10,11,12,13,14,15,16,17,18,19,20,21,22,23,24,25,26]. This multifunctional capacity is not unique to SARS-CoV-2 ORF3a but has been shown for other coronavirus accessory proteins, such as ORF3 from porcine epidemic diarrhea virus (PEDV), another proposed viroporin that shares many structural and functional features with ORF3a. Indeed, ORF3 from PEDV has been implicated in host immune evasion, vesicle formation, and modulation of apoptosis [49,50,51]. It is likely that any pathological effects from viral accessory proteins such as ORF3a or ORF3 are manifested through their multiple roles targeting various aspects of cellular physiology. Future studies may reveal how the accessory proteins may impact cellular physiology in a cell type- or tissue-specific manner. The coronavirus accessory proteins are important factors affecting viral virulence. Clearly there remain important knowledge gaps in our understanding of the function of these accessory proteins. Addressing such knowledge gaps will be crucial for guiding the design of therapeutics targeting coronavirus infection. 

## Figures and Tables

**Table 1:** X-ray diffraction data collection and refinement statistics.

	TRAF2 TRAF-C: ORF3a Peptide (8T5Q)	TRAF3 TRAF-C: ORF3a Peptide (8T5P)
Data Collection		
Spacegroup	C2	P1
Unit cell (a, b, c,) (Å) (α, β, γ) (˚)	135.20, 84.22, 124.39 90, 118.82, 90	82.35, 83.75, 83.80 60.03, 79.26, 83.80
Wavelength (Å)	1.033	1.033
Resolution (Å) (last shell)	29.61–1.90 (1.97–1.90)	41.62–2.50 (2.60–2.50)
No. of reflections (total/unique)	344,314/95,517	458,741/64,645
Completeness (%) (last shell)	98.6 (98.1)	97.9 (96.3)
<I/sigma(I)> (last shell)	12.19 (1.49)	11.11 (1.72)
R_meas_ (%) ^a^ (last shell)	7.2 (100.0)	17.4 (232.9)
R_pim_ (%) ^b^ (last shell)	3.8 (52.0)	6.5 (92.8)
CC ½ (%) ^c^ (last shell)	99.9 (70.3)	99.6 (50.5)
Multiplicity (last shell)	3.6 (3.7)	7.1 (6.3)
Refinement		
No. of non-H atoms (protein/solvent) ^d^	8109/396	9201/27
Rmsd bonds length (Å)/angles (˚)	0.007/0.947	0.008/0.925
R_work_/R_free_ (%) ^e^	20.7/24.0	19.7/24.2
Ramachandran plot (favored/disallowed)	98.51/0	96.92/0
Average B-factor (Å^2^)	43.34	62.56
protein	43.33	62.40
peptide	60.53	78.91
solvent	38.88	52.06

^a^ R_meas_ = Σ_hkl_ (n/[n − 1])^1/2^ Σ_i_ |I_i_(hkl) − <I(hkl)>|/Σ_hkl_ Σ_i_ I_i_(hkl), where I_i_(hkl) and <I(hkl)> are the ith and mean measurement of the intensity of reflection hkl, and n is the multiplicity. ^b^ R_pim_ = Σ_hkl_ (1/[n − 1])^1/2^ Σ_i_ |I_i_(hkl) − <I(hkl)>|/Σ_hkl_ Σ_i_ I_i_(hkl), where I_i_(hkl) and <I(hkl)> are the ith and mean measurement of the intensity of reflection hkl, and n is the multiplicity. ^c^ CC ½ = Σ (x − <x>)(y − <y>)/[Σ(x − <x>)^2^ Σ(y − <y>)^2^]^1/2^ where x and y are randomly split half data sets. CC ½ is the Pearson correlation coefficient of randomly split half data sets. ^d^ “Protein” includes TRAF and peptide atoms; “solvent” includes waters and polyethylene glycol (PEG) atoms (8T5Q only). ^e^ R_work_ = Σ_hkl_||F_obs_ (hkl)| − |F_calc_ (hkl)||/Σ_hkl_ |F_obs_ (hkl)|, where F_obs_ (hkl) and F_calc_ (hkl) are the observed and calculated structure factors, respectively. R_free_ is the R value obtained for a test set of reflections consisting of a randomly selected 5% subset of the data set which is excluded from refinement.

## Data Availability

The crystal structures described in this manuscript are deposited in the RCSB Protein Data Bank with accession codes 8T5P and 8T5Q.
